# Nano-Gels: Recent Advancement in Fabrication Methods for Mitigation of Skin Cancer

**DOI:** 10.3390/gels9040331

**Published:** 2023-04-13

**Authors:** Ghallab Alotaibi, Sitah Alharthi, Biswajit Basu, Dipanjana Ash, Swarnali Dutta, Sudarshan Singh, Bhupendra G. Prajapati, Sankha Bhattacharya, Vijay R. Chidrawar, Havagiray Chitme

**Affiliations:** 1 Department of Pharmaceutical Sciences, College of Pharmacy, Shaqra University, Al-Dawadmi Campus, Al-Dawadmi 11961, Saudi Arabia; 2 Department of Pharmaceutical Technology, Global College of Pharmaceutical Technology, Krishnagar 741102, West Bengal, India; 3 Department of Pharmaceutics, BCDA College of Pharmacy & Technology, Kolkata 700127, West Bengal, India; dipanjanaash16@gmail.com; 4 Department of Pharmacology, Birla Institute of Technology, Ranchi 835215, Jharkhand, India; swarnalidutta89@gmail.com; 5 Department of Pharmaceutical Sciences, Faculty of Pharmacy, Chiang Mai University, Chiang Mai 50200, Thailand; 6 S. K. Patel College of Pharmaceutical Education and Research, Ganpat University, Mehsana 384012, Gujarat, India; 7 Department of Pharmaceutics, School of Pharmacy and Technology Management, SVKM’s NMIMS Deemed-to-Be University, Shirpur 425405, Maharashtra, India; 8 Department of Pharmacology, Raghavendra Institute of Pharmaceutical Education and Research, Ananthapuramu 515721, Andhra Pradesh, India; 9 Faculty of Pharmacy, DIT University, Dehradun 248009, Uttarakhand, India

**Keywords:** mitogen-activated protein kinase, nano-gel, polymeric cross linked, thermodynamic stability, skin malignancies

## Abstract

In the 21st century, melanoma and non-melanoma skin cancers have become an epidemic outbreak worldwide. Therefore, the exploration of all potential preventative and therapeutic measures based on either physical or bio-chemical mechanisms is essential via understanding precise pathophysiological pathways (Mitogen-activated protein kinase, Phosphatidylinositol 3-kinase Pathway, and Notch signaling pathway) and other aspects of such skin malignancies. Nano-gel, a three-dimensional polymeric cross-linked porous hydrogel having a diameter of 20–200 nm, possesses dual properties of both hydrogel and nanoparticle. The capacity of high drug entrapment efficiency with greater thermodynamic stability, remarkable solubilization potential, and swelling behavior of nano-gel becomes a promising candidate as a targeted drug delivery system in the treatment of skin cancer. Nano-gel can be either synthetically or architectonically modified for responding to either internal or external stimuli, including radiation, ultrasound, enzyme, magnetic, pH, temperature, and oxidation-reduction to achieve controlled release of pharmaceuticals and several bio-active molecules such as proteins, peptides, genes via amplifying drug aggregation in the active targeted tissue and reducing adverse pharmacological effects. Several drugs, such as anti-neoplastic biomolecules having short biological half-lives and prompt enzyme degradability capacity, must be appropriate for administration employing either chemically bridged or physically constructed nano-gel frameworks. The comprehensive review summarizes the advancement in the preparation and characterization methods of targeted nano-gel with enhanced pharmacological potential and preserved intracellular safety limits for the mitigation of skin malignancies with a special emphasize on skin cancer inducing pathophysiological pathways and prospective research opportunities for skin malignancy targeted nano-gels.

## 1. Introduction

The concept “novel drug delivery system” (NDDS) refers to an innovative and rational approach based on physical (osmosis, diffusion, swelling, erosion, dissolution, and electron-chain transport) and bio-chemical (monoclonal antibodies, gene therapy, carrier loaded therapy, polymer-drug conjugates, liposome, and noisome) mechanisms that have been developed having optimized properties of drugs such as tunable particle size in micro and nano range, improved permeability parameters and specific site targeting via advancing our understanding of the pharmacokinetic and pharmacodynamic behavior of the drug. NDDS can be obtained via either of three ways the development of nano-carrier loaded, sustain released drug delivery systems, and application of micro-fluidics [1,2,3].

Nanoparticles have been revealed to be beneficial in gathering information at all phases of clinical trials due to their application in numerous innovative tests for the treatment and diagnosis of diseases. The advantageous characteristic of the nanoparticle is to allow multiple proteins to attach to their surface over conventional dosage forms [4].

**Figure 1:**
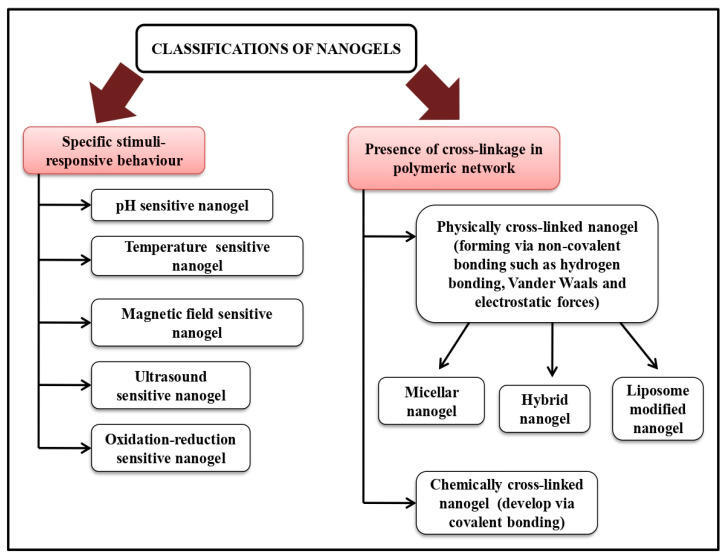
Classifications of nano-gels. Adapted from [25] under creative commons attribution 4.0 international license.

In 2008, Alexander and Serguei first coined the phrase “nano-particle constructed gel” to describe cross-linked bi-functional scaffolds of a swellable non-ionic polymer and a polyion for delivery of polynucleotides. Extremely cross-linked, three-dimensional nano-ranged hydrogel systems that can be either co-polymerized or made of ionic or non-ionic monomers are known as nano-gels having a size range of 20–200 nm, offering several beneficial effects such as having prolonged serum half-life, absorbing large amounts of body fluids without altering the fundamental networked structure and avoiding renal clearance [5,6]. The desire for nano-gels as a delivery system is fueled by their well-known excellent properties, including higher thermodynamic stability, remarkable solubilization potential, comparatively lower viscosity, and resistance to aggressive sterilization procedures [7]. Nano-gels, a dual combination of hydrogels and nanoparticles, have demonstrated significant promise as a targeted drug delivery system in the treatment of cancer, attributing to excellent drug loading capacity. Other advantages of nano-gel over other nanoparticles are to achieve active targeting via making a response to internal or external stimuli such as pH, temperature, light, and reduction-oxidation reaction with improved drug deposition in a targeted area, minimizing adverse effects of a drug via preventing drug accumulation in non-targeted tissues [8]. Nano-gels can be differentiated (Figure 1) into different categories such as physically-chemically cross-linked, hybrid nano-gel, polymeric, bio-mimetic nano-gel, pH, thermo, magnetic, hypoxia, ultrasound, enzyme, and reduction responsive nano-gel on the basis of synthesis procedure, nature of materials used and responsive towards stimuli respectively [8,9]. The swellable property of nano-gel makes them able to expand when in contact with physiological fluids providing flexibility to be near the targeted region drugs under particular physiological circumstances and increasing the dispersion of medicaments [10]. Nano-gels can be either synthetically modified to incorporate numerous ligands for targeted drug delivery or controlled drug release or primarily a transporter architecture for delivering pharmaceuticals and several bio-active compounds such as genes, proteins, etc. [11,12,13]. Depending on the type and properties of both drug and nano-gel being utilized as well as desired release pattern, there are many techniques to load drugs into them, such as physical entrapment (mixture of drug and nano-gel solution adsorb onto the surface or be encapsulated within the nano-gel matrix through physical entrapment), chemical conjugation (chemical reaction of drug molecules to nano-gel surface via covalent bonding), electrostatic interaction (electrostatic attraction between drug molecule and charged nano-gel surface) and stimuli (pH, temperature, and pressure) sensitive loading. However, the restriction in the entrapment of hydrophobic medications such as anticancer drugs in hydrophilic nano-gels can be overcome via modifying polymer structure, resulting in improved solubility and stability of poorly soluble drugs significantly [14]. Therefore, nano-gels are thought to be potential drug delivery carriers for the delivery and cellular uptake of proteins, peptides, and other biological compounds, attributing to relatively high affinity to aqueous solutions, remarkable thermal stability, bio-compatibility, fully or partially bio-degradability and suitability for molecular inclusion in bulk [15,16,17].

Skin disorders, the 4th foremost source of non-fatal diseases, are frequently the outward manifestation of more serious systemic illnesses, such as HIV, and neglected tropical diseases, such as elephantiasis and other ailments causing lymphedema [18]. Over a million cases of skin inflammation and cancer are discovered each year, making it by far the most prevalent form of cancer in people, especially in white people [19,20,21]. Skin malignancies are given names based on the cell from which they develop and their clinical characteristics. The three most frequent forms are cutaneous malignant melanoma, basal cell carcinomas, and squamous cell carcinomas. Basal cell carcinomas and squamous cell carcinomas are both also known as non-melanocytic skin cancers [19,22]. Similar to many other malignancies, Melanoma, the most combative skin cancer, greatly rises with age, likely due to the lengthy latency between carcinogen exposure and the development of cancer. In this case, UV exposure was a contributing factor [19,23]. In order to implement a coordinated and long-lasting global response to lowering the global burden, it is essential to comprehend the impact of dermatological disorders in wealthy countries around the world. Chemotherapeutic drugs cannot be used topically due to the physiological structure of the skin, poor affinity, and potency, whereas the drug-loaded targeted nano-gel formulations were found to be released more in slightly acidic melanoma micro-environment compared with other topical conventional formulations [24]. Therefore, nano-gels establish innovative architecture having improved pharmacological potential and maintained intracellular safety limits for the mitigation and treatment of targeted melanoma.

This review addresses fabrication techniques and characterizations of biocompatible nano-gels with an emphasis on the effectiveness of nano-enabled methods for the treatment of melanoma. Additionally, the framework explores potentials, challenges, and possible future advancements of nano-gel for the mitigation of skin malignancies.

## 2. Skin Cancer Pathogenies

**Figure 2:**
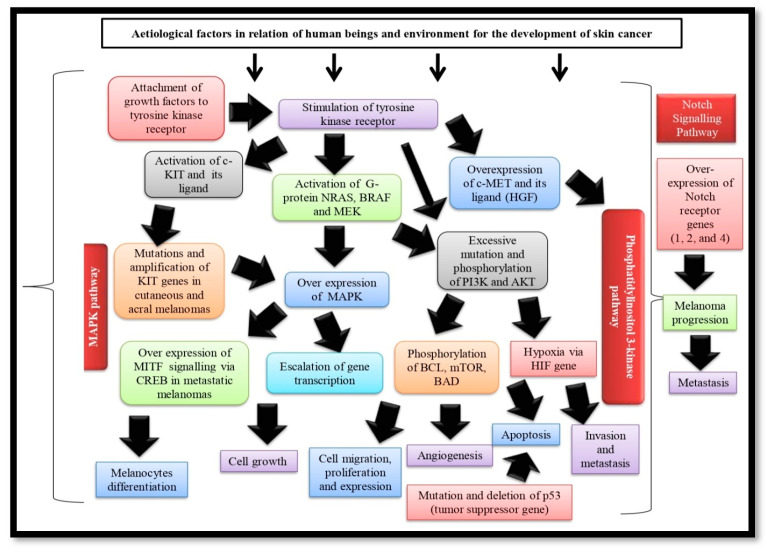
Pathogenesis, signaling and cellular pathways skin cancer. NRAS: Neuroblastoma RAS viral oncogene homolog. PI3K: Phosphoinositol-3-kinase AKT: Protein kinase B. mTOR: The mechanistic target of rapamycin BCL-2: B-cell lymphoma-2. BAD: Proapoptotic protein. NRAS—Neuroblastoma RAS viral oncogene homolog. BRAF: v-Raf murine sarcoma viral oncogene homolog B. MEK: Mitogen-activated protein kinase kinase. MAPK: Mitogen-activated protein kinase.

The distinguishing characteristic of melanocytes is the generation of melanin that itself possesses a complex feature of pro- and antioxidant [25,26]. Carcinogenesis initiates via the fundamental and first pathophysiological event, i.e., transforming antioxidant into a pro-oxidant agent in melanin under the influence of numerous pathogenetic stimuli related to human beings and the environment including long-term exposure to UV rays, skin phototypes, heredity (CDKN2A, CDK4, PTEN, etc.), immunosuppressive disorders, use of heavy metals, pesticides, etc. resulting in the damage of DNA molecule of melanocytes that raises intracellular oxygen radicals. These mutations cause uncontrollable cell proliferation, expression, and immortalization, attributing to the unrestricted stimulation of multiple cell signaling pathways [27]. The pathogenesis, signaling, and cellular pathways of melanomas (Figure 2) are described below:

### Mitogen-Activated Protein Kinase (MAPK) Pathway

Almost all forms of melanomas stimulate the MAPK cascade that controls cell proliferation, growth, and migration. Melanoma instances are characterized by excessive activation when the growth factors stimulate tyrosine kinase receptors after binding with it and resulting in the activation of the RAS family monomeric G protein, Neuroblastoma RAS viral oncogene homolog (NRAS) and serine/threonine kinases, v-Raf murine sarcoma viral oncogene homolog B (BRAF) BRAF. BRAF is over-expressed in melanocytes, brain tissue, testes, and hematopoietic cells, leading to phosphorylation and subsequent activation of MEK (the kinase of MAPK pathway), which in turn phosphorylates and activates MAPK and genes transcription factors that accelerate cell evolution, proliferation and migration [28,29]. The majority (70%) of BRAF gene mutations are caused by the conversion of thymidine to adenine, and the kinase domain of the protein molecule is activated as a result of the valine being switched out for glutamate (V600E). By interfering with the usual intramolecular interactions that keep BRAF in inactive configurations, these mutations indirectly cause BRAF activation [30,31]. Melanoma that develops in body regions exposed to sunlight is more likely to have the BRAF gene mutation (V600E) [29,31]. Other protein substitutions, available in V600K mutations (which account for around 20% of BRAF mutations responsible for early-stage melanoma development), are primarily observed in melanoma patients who have endured prolonged sun exposure. In 10% of the instances with radial growth and in 6% of in situ melanomas, BRAF mutations are less frequent in early-stage melanoma. These mutations do occur frequently observed in metastatic melanoma and are insufficient to trigger cutaneous melanoma alone. However present in around 80% of benign and premalignant lesions [30]. BRAF V600E mutations are more prevalent in women and have an inverse relationship to age, whereas BRAF V600K mutation rates rise with age [30,32]. Melanomas also exhibit enhanced cellular proliferation owing to mutations that result in increased RAS expression, but this occurs much less typically compared to other solid tumors. In melanoma, the most frequent NRAS mutation is Q16R [29]. The NRAS protein, which cannot “closed down”, may experience the heightened activity as a consequence of somatic NRAS gene mutations. Serine/threonine kinases are sequentially activated as a result, promoting cell cycle development, cell remodeling, and cell survival. The neurofibromatosis type 1 (NF1) tumor suppressor gene, which inhibits NRAS signaling, as well as the amplification and/or subsequent activation of a number of growth factor receptors, including c-MET, the epidermal growth factor receptor (EGFR) and c-KIT can also contribute to the series of events [33]. Only 10–20% of melanomas (particularly in amelanotic nodular variants) exhibited activating RAS mutations, with NRAS mutations becoming the most prevalent. Exceptionally frequent NRAS and BRAF mutations demonstrate that merely a single mutation in any one of the two genes is adequate to trigger the MAPK pathway [34]. The predominance of NRAS mutations is identified in congenital nevi, which are infrequent in nevi. Spitz nevi and NRAS mutations are typically linked [29]. Mutations in the NRAS or BRAF genes are found in 80% of cases of melanocytic nevi (40–50% and 15–20% mutations of BRAF and NRAS, respectively), highlighting the importance of MAPK pathway [30,35]. MAPK signaling governs Micro-phthalmia-associated transcription factor (MITF) activation, a key regulator necessary for myocyte maturation having an impact on the recurrence of some melanomas (particularly in metastatic melanomas). It has been revealed that the mutant oncogene BRAF can control the expression of MITF, ensuring that levels of protein are suitable for the growth and survival of melanoma cells [36,37,38].

## 3. Phosphatidylinositol 3-Kinase Pathway (PI3K) Pathway

PI3K-AKT, an important cell signaling cascade, can be physiologically triggered in carcinomas by either genetic alteration of PIK3CA and Protein kinase B (AKT1) or cognitive debilitation of phosphatase and tensin homolog deleted on chromosome 10 (PTEN). Tyrosine kinase receptor activation or RAS can either directly or indirectly activate phosphatidylinositol 3-kinase-lipid kinases (PI3 kinases), leading to the conversion of PIP2 to PIP3, which in turn phosphorylates AKT [30,35]. By changing PIP3 back to PIP2, PTEN lipid phosphatase plays a key antagonist in this pathway. Multi-factor proteins that control cellular activities (proliferation, viability, migration, revascularization, and metabolism) are phosphorylated as a result of AKT (serine/threonine protein kinase) phosphorylation. One of these proteins, called mTOR, has been discovered to be initiated in 73% of human melanoma cell lines and only extremely infrequently in neurons. Many melanomas have increased PI3K signaling, which is typically brought on by mutations, abolition, and stimulation of methylation of the PTEN inhibitor’s coding genes. Tyrosine kinase receptor c-KIT and stem cell factor (ligand of c-KIT) are critical for melanocyte maturation resulting in the deficiency of pigmentation and playing a role in the PI3K-AKT pathway [28,39]. Several immuno-histochemical research suggested the declination of c-KIT expression along with the progress of melanoma from a primary to a metastatic condition in acral melanomas and cutaneous melanomas in hands, feet, and nail beds (chronic sun-exposed areas) [34]. Tumor development is also associated with the amplification of the tyrosine kinase receptor c-MET along with its ligand HGF (hepatocytes growth factor), as numerous biological processes, including reproduction, viability, mobility, and penetration, are controlled by c-MET. Studies reported that over-secretion of HGF by melanoma cells or its micro-environment may cause the over-expression of c-MET, resulting in the formation of malignancies and metastasis via activating the PI3K-AKT pathway [30,40]. The main tumor suppressor gene, p53, is connected to apoptosis and responsible for a number of stressors, including DNA damage, genomic instability, hypoxia, and oncogenic aberrations. More than 50% of carcinomas have p53 mutations, including only 1–5% of original melanomas and 11–25% of metastatic melanomas; however, p53 is frequently lacking in nevi [41,42,43]. Hypoxia, a micro-environment of human skin, becomes a critical component in tumor genesis and metastasis, as typical hypoxia favors cellular proliferation [44]. Hypoxia-induced factor (HIF) is mainly responsible for mediating the hypoxia response resulting in the activation of numerous genes related to angiogenesis, invasion, and metastasis and promotion of PI3K pathway developing tumor and transforming melanocyte in the hypoxic human skin microenvironment. Due to inadequate vascularization, tumor hypoxia develops with melanoma growth, which raises HIF and promotes melanogenesis, and melanoma progression, having a detrimental prognostic impact [45].

## 4. Notch Signaling Pathway

The Notch signaling system, comprised of regulatory proteins, negative and positive regulators, a family of the receptor (four receptors in mammals) molecules, and their ligands (five ligands in mammals), plays a crucial role in the formation of tissue and homeostasis [46]. During metazoan development and in adult tissues, notch signaling is crucial for a variety of functions, including fundamental ones like cell differentiation determination, multiplication, and apoptosis [47]. Based on the type of cell, Notch can either stimulate or suppress the growth of tumors. Its stimulation by the excessive expression of Notch receptor genes 1, 2, and 4 contributes to the evolution and proliferation of melanoma, a process that is mediated by β-catenin [48,49,50]. Melanoma cells have increased Notch 1 signaling capable of converting a healthy melanocyte into a melanoma cell in the AKT pathway along with melanoma-inducing hypoxia [51]. The majority of Notch-mediated cellular effects arise from signaling pathways in which controlled proteolysis generates Notch transcription complexes governing the activation of regulated genes. Recently diverse Notch gene mutations were revealed in a variety of malignancies via unbiased genome-scale sequencing analysis. Interestingly, it was noted that the locations, characteristics, and outcomes of these mutations are cancer-specific and reflect various functions of Notch in different cancerous cells [52].

## 5. Method of Nano-Gel Preparations

Numerous techniques have been developed over the decades for the development of nano-gels based on excipients used. Nano-gels can be manufactured either concurrent polymerization-cross-linking process or stepwise [53,54].

### 5.1. Concurrent Polymerization and Cross-Linking

Nano-gels can be developed by carrying out cross-linking and polymerization simultaneously. Polymerization reactions are often conducted in aqueous media as the majority of aqueous soluble monomers and cross-linkers used in the synthesis of nano-gels are water-soluble. This simultaneous nano-gel synthesis process is classified into three categories such as precipitation polymerization, micro-template polymerization, and inversion emulsion polymerization, on the basis of their working principle.

#### 5.1.1. Precipitation Polymerization

The homogeneity is a key characteristic of precipitation polymerization reaction. In other words, prior to the reaction, all monomers, cross-linkers, and initiators are uniformly dissolved in the same reaction media homogeneously. After that, as the polymerization reaction develops, the polymer chain lengthens. The resulting phase is split out when the polymer chain reaches a specific length to create polymer colloidal particles, which then turn into nano-gels. Nano-gel size is governed by the number of monomers, cross-linking agents, and initiators. Nano-gels with physical cross-linking are supramolecular particles made of polymer molecules that were created by non-covalent interactions such as Ionic, hydrophilic-hydrophobic balance, Van der Waals, and hydrogen bonds [55]. The main advantage of the method is the absence of cross-linking agents, as the majority of cross-linking substances produce undesirable interactions when nano-gels are formed, and bioactive substances are enclosed. Drug loading in nano-gels can potentially be negatively impacted by cross-linking agents, which have been linked to severe side effects. In addition, by altering the physical conditions during formation, nano-gels can be made in any size for effective drug administration [10,56]. Kamenova et al. (2022) synthesized photo-chemically stable citric acid and pentane-1,2,5-triol loaded spherical and negatively (surface) charged nano-gel in tetrahydrofuran by precipitation esterification process in the presence of two catalysts (N-ethyl-N′-(3-dimethylaminopropyl) carbodiimide and 4 (dimethyl amino) pyridine). Later, doxorubicin hydrochloride was introduced into the physically cross-linked nano-gel because of electrostatic interaction between carboxylic groups in the nano-gel and amino groups of the anticancer drug exhibiting almost 95% entrapment efficiency with bi-phasic release kinetics [56]. Polymers created through covalent chemical interactions make up chemically cross-linked nano-gels. Low-molecular-weight monomers, polymer precursors, or polymers with particular terminal or pendular reactive groups are the starting materials [10]. Due to the stronger, irreversible bonds created by covalent interactions, the links established by chemical cross-linking techniques are more stable than those formed by physical procedures [55]. Polymerization by inverse emulsion polymerization, reversible addition-fragmentation chain transfer (RAFT), click chemistry cross-linking, and photo-induced cross-linking are some examples of chemical cross-linking techniques. The study reported that curcumin-loaded Carbopol, capmul (oil), Tween 20- polyethylene glycol 400 (surfactant) containing nano-gel was prepared by spontaneous emulsification technique for the treatment of squamous cell melanoma [57].

#### 5.1.2. Inverse Emulsion Polymerization

Nano-gels can be synthesized via suitable emulsification techniques using an oil-soluble emulsifier. The produced W/O inverse emulsions are capable of undergoing polymerization processes. Nano-gels that can be steadily disseminated in an aqueous media can be made after the removal of both components (organic solvent and emulsifier) [58]. Many variables, such as the choice of surface active agents, the quantity of both monomer and cross-linker and the pH of specific reaction media, all have an impact on the size of nano-gels [59]. The drawback of the method is that the reaction media is an organic solvent. The purification of the produced nano-gels will be problematic if emulsifiers or co-emulsifiers are present. A degradable poly (phosphorylcholine)-loaded (HPMPC) nano-gel was recently developed for drug release triggered by hypoxia in tumor cells. The produced nano-gel had a slow blood flow rate with a minute immunological reaction. The nano-gel showed efficient bio-degradability, bio-compatibility, and promising in vivo and in vitro tumor suppression effects [60].

#### 5.1.3. RAFT Polymerization

In Reversible Addition-Fragmentation Chain Transfer (RAFT) polymerization technique, a polymer (for example, Poly (N-vinyl caprolactam)) is subjected to a series of reactions (reversible addition and degradation of adducts with transferring chain of polymer) with dithioester compounds via regulating the molecular weight of the polymer throughout the free radical polymerization process. Amphiphilic polymers can change their micelle structures, including their length, orientation, and other characteristics, using RAFT technology [55,61]. Farmanbordar et al. (2021) developed a pH-responsive doxorubicin-loaded silica-polymethacrylic acid nano-gel via RAFT polymerization technique for the treatment of breast cancer [61].

#### 5.1.4. CCC Polymerization

Click Chemistry Cross-linking polymerization technique (including copper (I)-catalyzed azide-alkyne, copper-free, and pseudo-click chemical reactions) becomes a promising method for developing nano-gels attributed to its strong chemical reactivity, improved yield, and remarkable specificity [62]. For example, thiol-ene click chemistry, an efficient technique of developing doxorubicin-loaded pH-triggered nano-gels without byproducts via polymerizing methoxy polyethylene glycol acrylate, pentaerythritol tetra (3-mercaptopropionate) and ortho ester diacrylamide [63].

#### 5.1.5. PIC Polymerization

Photo-induced cross-linking (PIC) polymerization gains attention in the preparation of nano-gels due to its bacteriostatic action, excepient-free character, versatile functions, customizable particle diameter, and capacity to induce cross-linking. During this irradiation technique, water molecules break down into hydroxyl radicals and hydrogen atoms, having the ability to transform polymers into micro-radicals via intermolecular cross-linking, resulting in the development of nano-gels [64]. For instance, a photo-initiator-optical cross-linker (Irgacure 2959) was used in the development of docetaxel lipid-coated nano-gel [65,66].

### 5.2. Separate Polymerization/Cross-Linking

In this process, polymers are first synthesized, followed by the cross-linking of the polymeric molecular chains to form nano-gels. This approach is particularly appropriate for creating nano-gels from natural polymers [67]. The approach can be one of several different forms, including precipitation/cross-linking, emulsification/cross-linking, self-assembly/cross-linking, and micro-template forming/cross-linking, depending on the mechanism of nano-gel formation. Since the feature of the 3D network structure of nano-gels allows them to be carried with both hydrophilic and hydrophobic chemicals, nano-gels offer a platform for drug co-delivery. The study reported that phase-transition temperature emulsification was used to load doxorubicin and glycyrrhizic acid into alginate-based nano-gels, demonstrating synergistic anti-neoplastic effects mediated by both components of nano-gels in addition to their ability to target hepatocellular carcinoma [68]. The nano-curcumin and Sulph loaded ethosomal nano-gel were prepared using a combination of both thin-film hydration and phase transition temperature process [69].

Several pathogenic variables (environmental factors, UV exposure, immuno-suppression, and hereditary susceptibility) should be taken into consideration when designing bio-compatible, targeted, or sustained-release nano-gel without toxicity for the treatment of skin melanoma. For that purpose, one approach is to incorporate UV-blocking substances such as zinc oxide or titanium dioxide in the nano-gel matrix to protect from UV exposure. Another promising strategy for the treatment of skin cancer, immunotherapy, can be specially formulated to boost the immune system and improve anti-tumor response via incorporating immunomodulatory agents like interleukins, interferon, or checkpoint inhibitors in the nano-gel architecture [70].

## 6. Characterization of Nano-Gels for Skin Cancer Therapy

Owing to the tailored administration of bio-molecules with minimal toxicity, nano-gel is thought to be one of the most effective and appealing methods for treating skin cancer [71,72]. In designing the nanoparticle-based formulation, patient compliance is equally important [73]. Both in-vitro and in-vivo trials and tests have been conducted on these nanoparticle-based compositions [74]. Despite this, many kinds of research are still in the pre-clinical stage, and nanoparticle-based medicines have not yet received clinical approval for commercialization [75]. In this regard, we have highlighted characterization methods of topical nano-gel of anticancer medications (Table 1). 

### 6.1. Physico-Chemical Characterizations

Vesicle size, size distribution, zeta potential, and morphology of diluted nano-gel are analyzed by zetasizer and Transmission or Scanning electron microscope, respectively. Kriti et al. (2021) investigated the vesicle size of optimized nano-curcumin-DL-Sulforaphane-loaded ethosomal nano-gel in the range of 105.00 ± 5.00 to 152.00 ± 14.23 nm. Moreover, the optimized formulation’s polydispersity index and zeta potential values were found to be 0.296 ± 0.001 and −17.1 ± 2.61 mV, respectively, indicating a restricted particle size distribution and a high degree of uniformity [69]. The presence of ethanol in the formulation, which may release the negative charge from the surface of the vesicles, was the cause of the zeta potential on the ethosome. The stability of the vesicular system was generally improved by the negative surface charge on the ethosomal vesicle, which prevents the buildup of vesicles caused by resistive force and electrostatic repulsion [76,77]. TEM study on nano-ethosomal vesicles demonstrated size consistency and precise, spherical configuration [78]. Another study on curcumin nano-gel revealed zeta potential (−14.1 mV) and globule size (120–135 nm) via Malvern Zetasizer 90 and SEM, respectively [57]. Alhakamy et al. (2021) also observed well defined sealed, lamellar, spherical structure of brucine-loaded trans-liposomal nano-gel with uniform size distribution via Zetasizer instrument and TEM using the dynamic light scattering approach. The transmission electron micrograph also indicated the absence of crystalline brucine, confirming the complete entrapment of brucine inside the vesicular architecture [79].

### 6.2. Entrapment Efficiency and Drug Content Study

The nano-gels are diluted to get the required concentration using a suitable solvent, and the absorbance is measured at spectroscopy according to the monograph; therefore, the following formulas were used for the determination of entrapment efficiency and drug content.

$$Drug\;content=\frac{Ananlysed\;content}{Theoritical\;content}\times 100\;Percentage\;entrapped=\frac{Amount\;of\;drug\;entrapped}{Label\;claim}\times 100$$

Ganesh et al. (2019) found 96.8–98.8% curcumin in Carbopol and Tween20-PEG400 loaded nano-emulgel with satisfactory (96.2–99%) entrapment efficiency [57].

### 6.3. Applicability Parameter

The viscosity and pH of nano-gels are determined using Brookfield or Cone and plate viscometer at different rpm and electrometric methods, respectively, at room temperature. The viscosity (3152 poise) and pH (5.7) of curcumin nano-emulgel confirmed their suitability for topical application [57]. Moreover, texture analysis, including spreading co-efficient, firmness, cohesiveness, and consistency index, was also determined for topical nano-gel to commercialize the products. Curcumin-DL-Sulforaphane loaded ethosomal nano-gel presented firmness of 209.34 g, cohesiveness of −189.48 g, and consistency of 59.45 g/s, whereas brucine trans-liposomal nano-gel showed firmness (158.91 g), consistency (615.03 g/s) and cohesiveness (−115.26 g) substantiating topical or dermal delivery for skin cancer [79].

### 6.4. Swelling Study

The swelling property of both control nano-gel and drug-loaded nano-gel is examined at three different pHs (pH 4, 7, and 9) as the property of nano-gel may alter due to physical changes such as pH. Both nano-gels are converted to pellet form via a hydraulic pelletizer; therefore, the dry weight (W_o_) of the pellet is checked. Then the pellet was dipped in each pH solution for 5-min and again checked the weight of the wet pellet (W_w_) was as the surface of the pellet adsorbed the pH solution after removing the pH solution with filter paper. The following formula is used for the determination of the swelling capability of nano-gels.

Swelling ratio=(Ww−Wo)/Wo

Mangalathillam et al. (2012) observed swelling behavior of both control and curcumin-loaded chitin nano-gel at acidic, neutral, and basic pH and found more swelling of chitin nano-gel (both control and drug-loaded) at acidic pH in comparison to neutral and basic pH as chitin nano-gel, a polyelectrolyte nano-gel, may swell at a pH value below its pK_a_ and during exposure to acidic environment fixed charges are formed on the nano-gel framework. This work also revealed that the swelling ratio of curcumin-loaded chitin nano-gel is comparatively less than control chitin nano-gel at each pH condition attributing to the minimization of unoccupied reactive effective groups via curcumin amalgamation available in the chitin nano-gels [80].

### 6.5. In-Vitro Drug Release and Release Kinetics

The in-vitro drug release study is carried out in a Franz diffusion cell with phosphate buffer (pH 4.5 and 7.4) through a dialysis membrane at 37 ± 0.5 °C. A definite amount of nano-gel is placed on the Franz diffusion cell after being transferred to the cellophane membrane, and to keep and ensure the initial consistent volume, a specific volume of aliquots is taken out at intervals and replaced with an equal volume of fresh phosphate buffer. Thereafter the samples are examined using the specific spectroscopic technique as per the monograph [81]. The release kinetics modeling is done from cumulative percent release (CPR) data to explain the drug release mechanism, and the correlation coefficient (R^2^) value closest to 1 is selected to assess the best-fitted kinetic model. Kriti et al. (2021) observed about 89.33 ± 6.58% (at pH 4.5) and 80.67 ± 7.21% (at pH 7.4) of curcumin released from curcumin-DL-Sulforaphane loaded ethosomal nano-gel at 6 h. Curcumin released comparatively more in acidic pH, most presumably as a result of improved solubility of aggregated drugs within vesicles having relaxing integrity of nano-environment and swelling of Carbopol matrix became a promising candidate for skin cancer [69]. The drug release from curcumin ethosomal nano-gel followed Higuchi kinetics, having burst release initially following sustained apparent zero order release demonstrating a non-Fickian diffusion mechanism [82]. Another study reported that almost 80% of curcumin released from curcumin-loaded chitin nano-gel within 24 h at buffer solution (pH 4.5), whereas less than 30% of curcumin released at pH 7.4 buffer solution favoring tumor site-specific drug delivery [69]. In vitro drug release in open-sink conditions demonstrated a sustained daily release of 7–10% of loaded curcumin with a zero-order kinetics in PBS at 25 °C. Very sustained curcumin release was observed at pH 7.4 and 25 °C with the first-order kinetics of the daily 7–10% drug release [83]. A consistent release over an extended period was also observed to achieve a constant therapeutic benefit via controlling curcumin release rate from the multiple layer-by-layer (comprising of folic acid-casein coated carboxymethyl cellulose/casein) assembled nano-gel, resulting in the reduction of burst release initially and lowering systemic toxicity significantly. Moreover, relative to physiological pH 7.4, an elevated curcumin release profile was found at acidic pH (4.5 and 6.8), depicting the circumstances in endosomes and lysosomes where more quantity of curcumin would be taken up into the cell by receptor-mediated endocytosis [84,85]. Bagde et al. (2019) also reported that HPMC-based quercetin (0.12%)-titanium dioxide (15%) loaded nano-gels exhibited significant drug release (above 70%) compared to quercetin suspension at 24 h [85].

### 6.6. In-Vitro Skin Permeation and Dermatokinetic Study

The dorsal abdominal subcutaneous adipose skin layer of a rat with no visible signs of infection is removed, cleaned with isopropyl alcohol to remove adherent particles, and stored by wrapping in aluminum foil at −20 °C for studying in-vitro skin permeation. The preserved skin is mounted in the donor compartment of a Franz diffusion cell having 0.65 cm^2^ of surface area and 7.5 mL of volume operating in continuous stirring mode, and a definite amount of formulation is placed on top of it. For 24 h, the receptor media was maintained at a constant temperature of 37 ± 0.5 °C and a constant mixing speed in terms of rpm. To promote skin hydration, the donor compartment and receiver compartment solution are equilibrated for one hour. A specific quantity of aliquot is removed from the receiver compartment and replaced with fresh PBS at the designated times and therefore analyzed via spectroscopic techniques as per the monograph [86]. Studies showed significant (27.45 ± 4.55 µg/g weight of tissue) abdominal rat skin permeation. To get rid of any residual substance, the skin is rinsed with saline (pH 7.4) and then submerged for two to three minutes in warm water at 60 °C. Forceps are used to separate the skin sample’s epidermis and dermis layers. The removed skin layers are divided into small pieces and submerged in a suitable solvent for 24 h to extract the drug. Then the drug concentration is analyzed using a spectroscopic technique [69,87]. Separate curves are drawn between the drug content per cm^2^ of skin and time for the epidermis and dermis and assess T_skinmax_, C_skinmax_, AUC (0–8 h), and first-order excretion rate constant. The dermato-kinetic study revealed significant penetration of brucine from brucine-loaded trans-liposomal nano-gel [79,87].

### 6.7. Cytotoxicity Study

The cell viability assay of drug-loaded nano-gel, placebo, and control nano-gel formulations is carried out in cultured (in Dulbecco’s Modified Eagle’s Medium) murine melanoma cell lines (B16-F10). Therefore, the cultured cell is incubated at a temperature of 37 °C and relative humidity of 100% with 5% CO_2_. After treatment with 24 h, the cell lines are served with different concentrations of each formulation, and a specific quantity of MTT reagent [3(4,5-dimethylthiazol-2-yl)-2,5-diphenyltetrazolium bromide] is added into petri-dish after 48 h of and formulation treatment and again incubated for 4 h. Finally, a specific amount of DMSO is added to indicate purple color and absorbance is analyzed at a specific wavelength in Microplate reader. Alhakamy et al. (2021) reported a dose-dependent reduction in cell viability in brucine trans-liposomal nano-gel with an IC_50_ value of 37.23 μm, indicating the advancement of brucine delivery via nano-platform [79]. The MTT results also revealed that curcumin-loaded chitin nano-gel preserved the ability of curcumin to target melanoma specifically while sparing healthy cells, which is a crucial requirement for the cancer treatment strategy [83]. The IC_50_ value (13.1 µg/mL) of Curcumin loaded folic acid-casein coated carboxymethyl cellulose/casein nano-gel exhibited a comparatively significant cellular uptake profile than free curcumin (IC_50_ value: 18.3 µg/mL), attributing to folate-mediated targeting [84].

**Table 1:** Characterization of nano-gels for the management of skin cancer.

Nano-Gel	Particle Size (nm)	Zeta Potential	Comments	Reference
5-Fluorouracil-loaded chitin nano-gel	125–140 nm	+31.9 mV	The relaxing of keratin and the deposition of 5FU in the deeper layers of skin were eventually caused by the positive zeta potential of chitin nano-gel facilitating the establishment of a strong association with stratum corneum.	[88]
Nano-curcumin and sulforaphane loaded ethosomal nano-gel	125.67 ± 10.43 nm	–17.1 ± 2.61 mV	The excellent cytotoxicity against the B16-F10 murine tumor cell line and the remarkable radical scavenging effect of optimized ethosomal nano-gel confirm effectiveness in the treatment of melanoma.	[78]
Curcumin loaded Carbopol nano-emulgel (containing Capmul: Tween20-PEG400 = 1:8)	125.3 nm	−14.1 mV	The significant, sustained curcumin release from optimized nano-emulgel with enhanced permeability and less toxicity assures a promising candidate for the treatment of squamous cell carcinoma	[57]
Curcumin-chitin nano-gel	70–80 nm	+49.34 mV	The satisfactory particle size, drug entrapment, release capacity, surface characterization, and excellent skin penetration property of curcumin-loaded chitin nano-gels became a potential candidate for the mitigation of skin cancer via transdermal route	[83]
Brucine-trans-liposomal nano-gel	136.20 ± 2.87 nm	-	The optimized trans-liposomal nano-gel formed depots in the deeper layers of the skin via continuous release of brucine for a prolonged period of time, minimizing the dosage frequency	[79]

## 7. Nano-Gels in Skin Cancer

**Figure 3:**
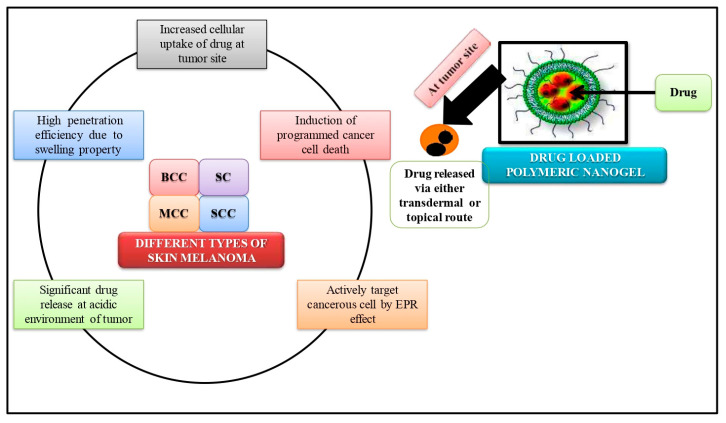
Mechanisms of polymeric nano-gels in the treatment of skin cancer. BCC: Basal cell carcinoma, SCC: Squamous cell carcinoma, SC: Superficial carcinoma, MCC: Merkel cell carcinoma.

In order to overcome the limitations of traditional anticancer therapies and enhance the outcomes of cancer treatment, nano-gels have been demonstrated to be one of the best solutions. The fundamental drawback of traditional chemotherapy is that it targets both diseased and non-cancerous cells through a non-selective process. Increased toxicity and negative impacts result from this. In order to deliver chemotherapeutic medications to cancer cells with low side effects and reduced toxicity, stimuli-sensitive nano-gels (Figure 3) have been effectively developed and assessed [89]. Hormone therapies are effective for tumors linked to hormones [90] but several hormonal therapies have been linked to elevated risk factors for diabetes mellitus and thrombosis [91]. Nano-gels designed for targeted medication delivery in these cancer types are not linked to such risk factors. The most prevalent type of cancer in people is skin cancer. 

“Skin cancer” refers to a variety of medical conditions that develop from distinct epidermal and dermal cells. Skin cancer has been successfully treated with loaded nano-gels of chitin-polymerized curcumin [83]. The therapeutic effectiveness of the B16-F10 melanoma tumor was improved by a thermo-sensitive nano-hydrogel co-loaded with DOX, IL-2, and IFN-γ, which boosted tumor cell death and enhanced replication of CD3+/CD4+ and CD3+/CD8+ T cells [89]. Drugs having high molecular weight have been formulated as nano-gels to enhance their solubility. For instance, tacrolimus, an immune-suppressive drug, was loaded in polyglycerol polymerized thermo-sensitive topical nano-gel and found to be improved adsorption of the drug through the layers of skin, attributing to the elevated body temperature for inflammation resulting in remarkable anti-proliferative activity [92]. pH-responsive biodegradable 5-fluorouracil-loaded chitosan nano-gel was developed to treat melanoma in the mild acidic cancer micro-environment, effectively preserving the integrity of the skin layer in comparison with other traditional melanoma formulations [93]. Transdermal curcumin nano-gels were developed to improve the solubility, transdermal permeability, and significant release of curcumin with lower toxicity attributed to their physicochemical characteristics when compared to traditional curcumin formulations [57]. In order to create hydrophilic and thermo-responsive three-dimensional crosslinked nano-gels that can improve the penetration of both smaller and larger molecules through the squamous cells and aggregate within the hair follicles, dendrimers made of dendritic polyglycerol (dPG) were utilized [15,94,95,96,97,98]. After interacting with the squamous cell, such nano-gels can be designed to go through a physical transition for improved dermal penetration and payload release in response to the ionic strength, temperature, or pH gradient of the skin [92,99]. For instance, chitosan or PLGA-chitosan nano-gels that are pH-responsive and biodegradable have demonstrated the capacity to release 5-FU in reaction to the tumor’s acidic environment to cure melanoma [93,100]. Nano-gels made of chitin have also demonstrated the capacity to carry medications deep within the skin, treating inflammatory conditions like psoriasis [101]. Another study reported that pH-sensitive biodegradable, bio-compatible, cytocompatible, self-assembled, and chemically cross-linked chitosan-Pluronic 127 loaded nano-gel exhibited pH-triggered bleomycin release in a sustained manner to the cutaneous area having significant entrapment efficiency (55%), providing a unique strategy against skin melanoma [100]. A chemo-preventive study revealed that the average integer of UVB-inducing tumor, tumor volume was less in a remarkable manner in the quercetin-titanium dioxide nano-gel treated animals with improved quercetin deposition on the skin via down-regulating COX-2, EP3, EP4, PCNA, and cyclin D1 expressions [85]. Therefore, for applications in targeted medication delivery, diagnostics, bio-sensing, and the separation of biological constituents, nano-gels have gained significant research interest.

## 8. Overview of Patent Situation

**Figure 4:**
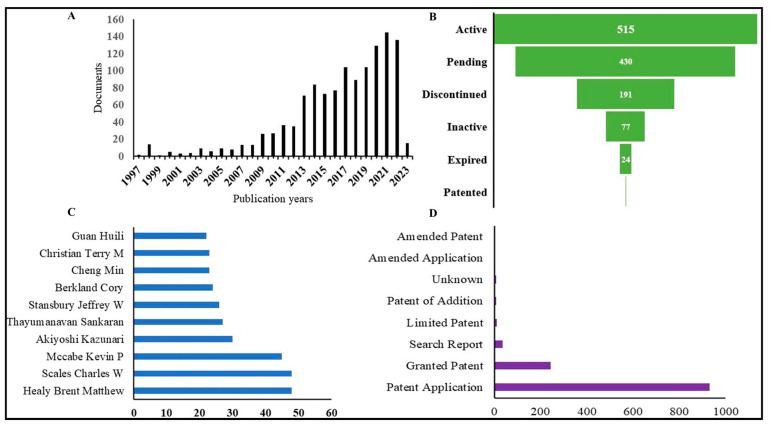
The patent situation on nano-gels in the management of skin cancer. (**A**) Patents applied year wise (**B**) Legal status of patents applied (**C**) Major Innovators applied for patents (**D**) Document type (Granted and applied data).

Due to its distinctive features, nano-gel is increasingly being recognized as significant material for a variety of applications. Nano-gels can be used in different branches of science like drug delivery, tissue engineering, cosmetics, etc. Patent search with keywords “Nano-gel” and “Nano-gel” + “Skin Cancer” in the title, abstract, and claim showed 1238 and 9 patent results, respectively (Table 2). The patent export data of nano-gel indicate that in recent years the number of patents increasing drastically to more than 100 per year (Figure 4A) with application fields like cellular, ocular, dental, and skin delivery of therapeutics and biologicals. A new class of research includes 3D-printed polymeric nano-gel particles. Out of 1238 patents on nano-gel, around 500 were active, and 400 were pending status (Figure 4B); the United States of America with the highest number of 406 applications, followed by China with 332. Figure 4C lists the major inventor in this field, and Figure 4D shows that to date, 253 patents have been granted and 922 patent applications.

## 9. Future Prospects and Conclusions

The possible development of nano-gels holds out considerable promise and provides researchers with new chances for medication delivery against a wide range of diseases and ailments. We can achieve optimal drug loading in the nano-gels by modifying the nano-gel system and precisely adjusting its composition, such as polymer type, molecular weight, and cross-linking density. In addition, other functional components like imaging probes and targeting moieties can be easily bonded onto nano-gels to investigate their potential for tailored therapeutic effects. Because of their multi-functionality, 3-dimensional polymer construction, and stimuli-responsive qualities, the ideal nano-sized properties of these agents would make nano-gels very appealing for topical targeted cancer therapy with minimal toxicity burden on patients. Nano-gels have the potential to open the door to effective target medication delivery to tissues and cancerous cells. Many factors, including those relating to patients and drugs, interact during cancer chemotherapy. Overall, in vitro and in vivo stability of dosage forms is crucial to ensure that it reaches the target site undamaged. When administered intravenously, which is the primary method of delivery for most cancer medications, nano-gels have shown to be a very stable dosage form that can deliver the required payload to malignant areas. As biocompatible biomimetic hydrogels are predicted to avoid the reticuloendothelial system and change the nuclear phagocytic system, they are likely to play a substantial impact on intracellular drug carriers resulting in a prolonged in vivo circulation period. Hence, drug delivery investigators must concentrate on these new kinds of nano-gels while also utilizing biocompatible, stable, and biodegradable natural polymers. The usage of polymer hybrids, or molecular blends made of a combination of synthetic and natural polymers, is another feature of nano-gels that will catch the interest of formulation scientists in the ensuing 10 years. Natural polymers would have a beneficial impact on biocompatibility and biodegradability, while synthetic polymers would offer more functionality. Natural polymers would provide an excellent candidate for implant delivery systems for anticancer medications because they do not produce hazardous breakdown products, in contrast to some synthetic polymers. When nano-gels are implanted into malignant regions, drug release will take place as expected owing to the engineering techniques used during formulation. This technique of delivery will significantly lessen off-target toxicities linked to the systemic distribution of various anticancer medicines by reducing drug migration to undesirable regions. The potential for one of the most significant breakthroughs in targeted cancer therapy has been expanded owing to nano-gel formulations of anticancer medications. Future applications of nano-gels in targeted cancer therapy have shown considerable promise in preliminary studies. For nano-gel formulations, there is a need to standardize procedures with a good manufacturing practice manual. In order to establish the safety profile of nano-gels and further our understanding of them, it is crucial to scale up research on nano-gels for targeted cancer therapy. The potential for using nano-gels as a target delivery method for anticancer medications is still quite great, and emerging pharma companies are anticipated to take advantage of this potential to improve the translational research of these medications.

## Figures and Tables

**Table 2:** Patents on Nano-gel for Skin Cancer.

Application Number/Display Key	Publication Date	Title	Legal Status	Reference
US 202017599948 A/ US 2022/0195071 A1	23 June 2022	Immunotherapeutic compositions and use thereof	Pending	[102]
US 2020/0025844 W/ WO 2020/205808 A1	8 October 2020		Pending	[103]
EP 20210058 A/ EP 4005577 A1	1 June 2022	Cellular and/or extracellular extracts for preventing and/or treating cancer and/or inflammation	Pending	[104]
EP 2021083238 W/WO 2022/112528 A1	2 June 2022		Pending	[105]
EP 2020083705 W/WO 2021/105407 A1	3 June 2021	Mirna-based pharmaceutical compositions and uses thereof for the prevention and treatment of tissue disorders	Pending	[106]
EP 2020083702 W/WO 2021/105404 A1	3 June 2021	Biomaterials for the prevention and treatment of tissue disorders	Pending	[107]
US 201715791999 A/US 2018/0085479 A1	29 March 2018	Cathepsin-binding compounds bound to a carrier and their diagnostic use	Active	[108]
EP 11005110 A/ EP 2537532 A1	26 December 2012	Cathepsin-binding compounds bound to a nanodevice and their diagnostic and therapeutic use	Discontinued	[109]
US 2021/0056621 W/ WO 2022/093800 A2	5 May 2022	Carrier particle-drug conjugates, self-immolative linkers, and uses thereof	Pending	[110]

## Data Availability

Not applicable.
